# Relationship between Upper Quarter Y Balance Test performance and throwing proficiency in adolescent Olympic handball players

**DOI:** 10.1186/s13102-020-00199-4

**Published:** 2020-08-27

**Authors:** Julian Bauer, Simon Schedler, Stephan Fischer, Thomas Muehlbauer

**Affiliations:** Division of Movement and Training Sciences/Biomechanics of Sport, University of Duisburg-Essen, Gladbecker Str. 182, 45141 Essen, Germany

**Keywords:** Postural control, Upper quarter mobility/stability, Throwing velocity/accuracy, Young athletes

## Abstract

**Background:**

Olympic handball is a sport mainly focused on executing throwing and passing techniques with the throwing arm. Functional specialization due to the unilateral characteristic and dominance of the throwing arm may lead to adapted control of shoulder stability and mobility. Thus, we examined side differences between the throwing and the non-throwing arm. Additionally, correlations between the Upper Quarter Y Balance Test (YBT-UQ) and handball-specific performance measures were investigated.

**Methods:**

All participants (F = 13 yrs., *n* = 14, training experience [te] 5.9 ± 1.3 yrs.; M = 14 yrs., *n* = 24, te 6.5 ± 2.5 yrs.; M = 15 yrs., *n* = 18, te 9.3 ± 2.2 yrs) were Olympic handball players of a regional youth selection team. YBT-UQ was executed assessing performance in medial, inferolateral and superolateral reach direction normalized to the upper limb length together with a composite score of the mean of all reach directions. A radar gun and a target net were used for the assessment of throwing velocity and throwing accuracy. The paired *t*-test was used to detect side differences in YBT-UQ performance. Pearson’s correlation analysis was calculated for associations between YBT-UQ and throwing velocity/accuracy.

**Results:**

Significant differences between the throwing and non-throwing arm were only detected for the superolateral reach direction in the 14-year-old males. Small correlations between YBT-UQ performance and throwing velocity/accuracy (13-year-old females: − 0.01 ≤ *r* ≤ − 0.37 / 0.01 ≤ *r* ≤ 0.31; 14-year-old males: 0.10 ≤ *r* ≤ 0.45 / -0.01 ≤ *r* ≤ .-0.51; 15-year-old males: 0.06 ≤ *r* ≤ 0.34 / 0.01 ≤ *r* ≤ − 0.45) were observed, irrespective of age and sex category.

**Conclusions:**

There was only a minimal difference in performance of the YBT-UQ between the throwing and non-throwing arm and only weak if any relationships between throwing performance and stability/mobility of the upper extremities in adolescent Olympic handball players existed. Further research is needed to investigate whether the YBT-UQ is a useful tool to detect training-related improvements in measures of shoulder stability/mobility and functional performance.

## Background

Olympic handball is a fast-paced contact sport [1]. Contrary to soccer in which bilateral exercises are frequently used in systematic training [2], Olympic handball training is mainly focused on executing throwing and passing techniques with the throwing arm, with the athletes rarely varying the playing arm and hand. Therefore, the functional specialization due to the unilateral characteristic and dominance of the throwing arm may lead to adapted movement control [3, 4] following the regular and specific unilateral execution of Olympic handball training.

Throwing is a key factor in Olympic handball [5, 6] and a complex skill including motion technique [7], physical characteristics [8], and motor skills [9, 10] involving both mechanical and muscular aspects [11]. Throwing techniques can be divided into the standing throw, the standing throw with up to three steps run-up and the jump throw [11]. A proximal to distal sequential muscle activation [12, 13] and the need for an effective energy transfer [14] is present in all these types of throws. Beside the different throwing techniques, optimal scapula control [15] and the need for thoracic mobility [16] seem to be of high importance in throwing. More specifically, shoulder internal rotation, a functional elbow angle [9] as well as elbow extension [11] may play an important role in throwing velocity. Consequently, mobility in combination with segmental stability as assessed through the Upper Quarter Y Balance Test (YBT-UQ) may be of high importance for throwing proficiency development. The YBT-UQ has been shown to be a reliable and valid [17–19] closed chain test for the assessment of upper quarter mobility and stability. The YBT-UQ was performed by every subject demanding closed chain stability [17, 19–21] in all three reach directions tested, i.e. medial (MED), inferolateral (IL), and superolateral (SL).

At this time no studies have investigated adolescent Olympic handball players and their functional adaptation to a sport with a strong unilateral execution component in the upper extremities. The YBT-UQ assessment will be used as a possible test that could correlate with Olympic handball-specific performance measures and to further detect probable side differences. Thus, the primary purpose of the present study was to examine whether there are side differences between the throwing and non-throwing arm. A second aim was to assess the relationship between the YBT-UQ and Olympic handball-specific performance measures. These findings may help practitioners and scientists alike to assess the importance of training programs to decline dysfunctional asymmetries and to avoid decrements in throwing performance as the most important technique in Olympic handball. Additionally, the results may give insight into the demand to develop specific functional upper quarter mobility and stability programs in order to improve Olympic handball-specific throwing performance.

## Methods

### Subjects

All subjects (*n* = 14 female aged 13 years, *n* = 24 males aged 14 years, *n* = 18 males aged 15 years) were Olympic handball players of a regional youth selection team of the Handball Association Niederrhein. The subjects were recruited due to their belonging to a regional youth selection team with a similar training regimen (i.e., training frequency of 3–4 times per week), playing level (i.e., regional) and age span (i.e. adolescence), also being comparable for both sexes. All subjects and their parents or legal guardians were informed about possible risks, the study’s objectives, and testing procedure. Further, a video was send to their coaches 1 week before the testing to demonstrate the YBT-UQ. Written consent of all subjects and an informed consent of the parents or legal guardians was obtained prior to the testing. Exclusion criteria were any injury that prevented the subjects from training or playing in the 2 weeks prior to the testing as well as vestibular, visual or proprioceptive disorders and functional limitations that were judged as possibly affecting YBT-UQ or throwing performance. Prior to the first training participation of the selection team, all athletes had to complete a questionnaire about their medical and clinical status including any current medications. Besides the written consent and medical clearance to take part in the training of the team, every athlete had to present a mandatory cardiac screening for Olympic handball youth players, which was controlled for by the Olympic handball association. The study was carried out according to the declaration of Helsinki. Additional characteristics can be found in Table 1.

**Table 1:** Characteristics of the study participants (*N* = 56) by group

Characteristic	F: 13 yrs. (*n* = 14)	M: 14 yrs. (*n* = 24)	M: 15 yrs. (*n* = 18)
Body height (cm)	165.4 ± 6.2	178.3 ± 7.6	181.4 ± 8.0
Body mass (kg)	58.9 ± 12.2	67.8 ± 13.2	69.8 ± 10.1
Body mass index (kg/m^2^)	21.4 ± 3.5	21.2 ± 2.7	21.2 ± 2.4
Left arm length (cm)	83.4 ± 4.3	89.9 ± 4.0	92.7 ± 4.1
Right arm length (cm)	84.5 ± 4.4	90.6 ± 4.1	93.3 ± 4.0
Arm dominance (L/R)	1/13	3/21	3/15
Throwing arm (L/R)	0/14	2/22	2/16
Training experience (yrs)	5.9 ± 1.3	6.5 ± 2.5	9.3 ± 2.2

Note. Values are mean ± values standard deviations. *F* Female; *L* Left; *M* Male; *R* Right

### Testing procedures

#### Measurements

The testing was carried out on three different measurement days. All testings were executed at the same time in the evening in the training venue of the Olympic handball association. Testing personnel, which consisted of experienced raters, was identical for all three testings at every station. All subjects were randomly assigned to one of the three testing groups. All groups started at the same time with group 1 starting with the anthropometric assessment, group 2 starting with the YBT-UQ testing and group 3 starting with the throwing velocity and accuracy testing. Following each station, the groups were given a 5-min break to avoid fatiguing effects for the next station. A standardized verbal instruction was given prior to each test and a standardized warm-up was executed including 5 min of submaximal running followed by a mobility routine consisting of different functional exercises, and working with rubber bands as stretching was not permitted. Additionally, different standardized passing techniques (with Olympic handballs with the according throwing size 1 or 2, depending on female or male subjects) were executed prior to the throwing attempts.

#### Assessment of anthropometric characteristics

Upper limb measurements were carried out with a tape measure from the seventh cervical spinous process to the distal tip of the middle finger of the arm with the shoulder in 90° of abduction [22]. Measurement of body mass was assessed with a Seca clara 803 digital scale. Standing body height was assessed with a Seca linear measure scale without shoes. The subjects were asked to lean against the scale with their feet on the ground, looking forward and straight. The according height was determined in centimetres from the ground to the top of the subjects` head. Additionally, the subjects were asked for how many years they had been training and playing Olympic handball, which position they played and to identify their dominant and throwing arm.

#### Assessment of upper quarter Y balance test performance

**Fig. 1:**
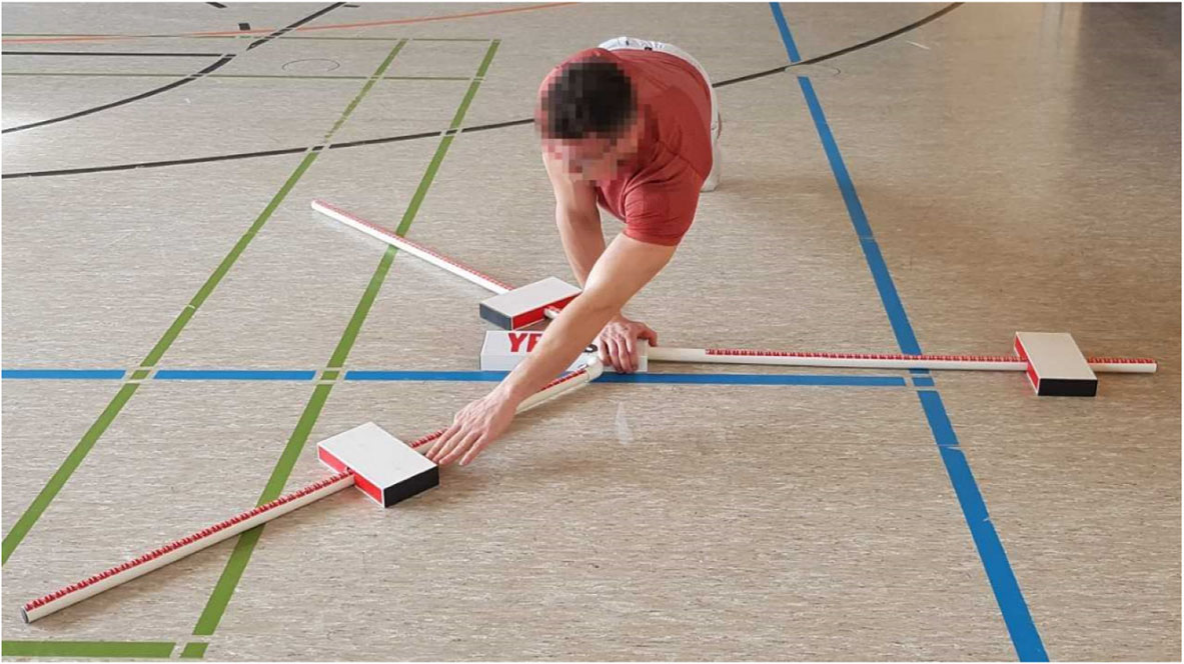
Participant performing the YBT-UQ in superolateral reach direction

A Y Balance Test Kit (Move2Perform, Evansville, IN) was used together with an adapted YBT-UQ testing protocol. Standardized verbal instruction was given to all participants prior to executing the test and one of the experimenters performed a demonstration trial before the testing. All participants assumed a push-up position with their feet shoulder wide apart [22]. Out of this position, the participants moved the indicator with their free hand into the MED, IF, and SL directions (Fig. 1). All three reach directions had to be performed without any break and while maintaining the push-up position with the contralateral arm. The trial was stopped if the subject was either not maintaining the push-up position, dynamically pushing the indicator box out of his/her reach or lost three-point contact on the surface. After every complete trial, a 30-s rest period was granted before completing the next trial. When all three trials beginning with the right arm as the stance arm were completed, every subject performed the testing with the opposite limb with the same breaks as during the first trials. Each score was recorded for every reach direction and the best score was taken into consideration for the analysis [22]. The composite score (CS) was calculated as the mean of the averaged maximal distances in all three reach directions [23]. Additionally, all reaches were normalized for upper limb length.

#### Assessment of throwing velocity and accuracy

**Fig. 2:**
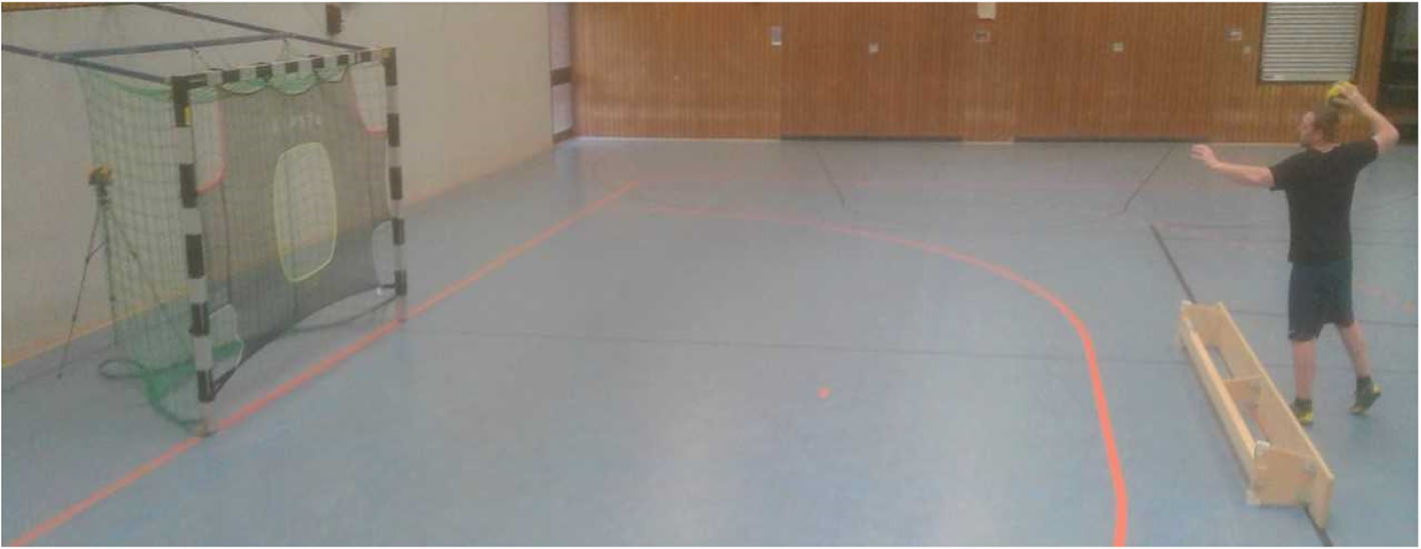
Goal net with radar gun behind the posterior goal net

A target net (SG 500 L; size: 3 × 2 m) was attached to a standard Olympic handball goal (Fig. 2). As scoring success is highly dependent on accuracy and velocity measures were taken of both. Previous studies [24–26] have found that there is a relationship between accuracy and velocity in Olympic handball throws and that an increase in velocity is not necessarily followed by a decrease in accuracy [11, 25, 27] contrary to the speed-accuracy trade-off reported by other authors [28]. To measure throwing velocity a „Stalker Pro “radar gun (Applied Concepts Inc., 2609 Technology Drive, Plano, TX 75074–7467, USA) was used. The Doppler radar gun is reported to be a highly reliable instrument for the assessment of throwing velocity with an ICC between 0.97 and 0.98 [29]. The „Stalker Pro “is able to measure velocities from 0 to 480 km/h with an accuracy of 0.16 km/h in a time interval of 0.01 s. The working frequency is reported to be 35.1 GHz with a low disturbance threshold. The radar gun was positioned 1.5 m behind the goal net in the height of 1.2 m in the direction of throwing to secure the Doppler effect and the right detection angle of throwing. To guarantee the correct assessment of the throwing velocity, a second radar gun was positioned behind the thrower. In case a value was not assessed by the first radar gun behind the goal net, the value of the second radar gun which was the same model, would have been taken. One tester positioned himself behind the radar gun, while a second one was positioned behind the thrower with the second radar gun, with the third tester noting down the values into the scoring sheet.

Each subject stood with the contralateral foot in front of their throwing arm positioned at the 7-m line. A bench was put alongside the 7-m line (Fig. 2). The contralateral foot was allowed to touch the bench, but the participants were not allowed to fall over the bench following the trial. The players had to use a throwing technique with the elbow at or above shoulder height. In case a player did not follow this requirement and threw under shoulder height, the trial had to be repeated. In line with the testing procedure of the German Handball Federation [30], the female athletes used a standard ball size 1, while the male athletes used a standard ball size 2. The use of glue or resin was allowed to simulate a training or game-like situation as glue or resin is usually used during training or games on this competitive level. A standardized instruction was given prior to each task. As the first task, the subject had to throw the ball in the direction of the middle hole with maximal velocity. Each subject had three consecutive trials of which the one with the highest velocity was recorded. Between each trial, a short rest was permitted and the subjects executed the next throw based on their own perception of readiness. The test-retest reliability for the test of throwing velocity was reported to be *r* = 0.83 [30]. As throwing and passing during games are primarily executed unilaterally with the dominant arm, only the throwing arm was tested. After all subjects executed the first task of throwing with the highest velocity possible, they were instructed to throw into the middle hole with the additional information that only the successful throws into the middle hole, i.e. accuracy was counted regardless of velocity with each successful throw being awarded one point. Again, every subject had three consecutive trials with a short rest to take the next ball after each trial, making it possible to achieve three points in total. For that task, every successful trial was counted by the testing personnel. All three raters observed if the throws were passing through the correct hole. As a third task, the subjects had to throw into one of the four corners (Fig. 2). Every subject could decide for themselves which corner they used as a target and the same corner was allowed as a target for all three trials. As throws at the four corners have a lower scoring probability [31], it was decided to award successful throws at the centre with one point and successful throws at one of the corners with three points, making it possible to achieve nine points in total while velocity was not measured. The total score was calculated after the two tasks with the first one leading to 0–3 points and the second one leading to 0–9 points. Therefore, scoring of both scoring tasks together could lead to up to twelve points in total.

### Statistical analysis

Descriptive statistics (i.e., means and standard deviations) were calculated for each group with SPSS version 24.0 (IBM Corporation, Armonk, NY, USA). Further, comparisons for YBT-UQ performance and throwing proficiency between the throwing and non-throwing arm were executed using paired *t*-tests. A *p*-value of < 0.05 was considered statistically significant. In addition, Pearson’s correlation coefficient *r* was calculated for associations between YBT-UQ performance and throwing velocity. Based on the recommendations of Vincent [32], values of 0 ≤ *r* ≤ 0.69 indicate small, 0.70 ≤ *r* ≤ 0.89 medium, and *r* ≥ 0.90 large sizes of correlation.

## Results

Table 2 illustrates mean values and standard deviations for the normalized YBT-UQ performance by group. We did not detect significant differences for any of the reach directions or the CS between the throwing and non-throwing arm, except for the SL reach in the 14-year-old males (i.e., in favor of the non-throwing arm: *p* = 0.029). YBT-UQ performance was greatest for the MED reach direction, followed by the IF and the SL reach directions.

**Table 2:** Upper Quarter Y Balance Test performance (% arm length) by group

Group	F: 13 yrs. (*n* = 14)			M: 14 yrs. (*n* = 24)			M: 15 yrs. (*n* = 18)		
Arm	T arm reach	NT arm reach	*p*-value	T arm reach	NT arm reach	*p*-value	T arm reach	NT arm reach	*p*-value
MED	111.8 ± 10.0	111.8 ± 7.3	1.000	112.4 ± 7.6	112.7 ± 6.9	.805	111.2 ± 10.6	110.6 ± 10.6	.739
IF	98.9 ± 11.0	98.2 ± 12.8	.842	108.8 ± 12.1	106.9 ± 12.2	.379	106.0 ± 10.6	104.8 ± 8.1	.555
SL	79.4 ± 8.6	78.1 ± 7.3	.573	82.5 ± 8.8	79.9 ± 9.4	.029	79.1 ± 11.6	80.0 ± 10.0	.733
CS	96.7 ± 7.1	96.1 ± 7.5	.633	101.1 ± 7.5	99.7 ± 7.1	.170	98.7 ± 8.5	98.3 ± 7.3	.820

Notes. According to Plisky [33] the arm that is being measured is the stance arm (i.e., T arm reach means that the NT arm is measured). Values are mean ± values standard deviations. *CS* Composite score; *IF* Inferolateral; *M* Male; *MED* Medial; *NT* Non-throwing arm; *F* Female; *SL* Superolateral; *T* Throwing arm

Throwing performance is shown in Table 3. Based on the values of the German Handball Federation [30] which categorizes throwing velocities into the five categories, i.e., high above average, above average, average, below average, and highly below average – every individual group was ranked (F = 13 yrs., M = 14 yrs. and M = 15 yrs) in the category “average”.

**Table 3:** Throwing performance by group

Outcome	F: 13 yrs. (*n* = 14)	M: 14 yrs. (*n* = 24)	M: 15 yrs. (*n* = 18)
Velocity (km/h)	67.2 ± 4.7	85.9 ± 7.4	85.5 ± 7.2
Accuracy (pts.)	7.5 ± 3.0	5.0 ± 3.1	6.3 ± 3.2
Velocity categorization			
High above average	0	0	0
Above average	3	8	4
Average	10	14	12
Below average	1	1	2
Highly below average	0	1	0

Note. Values for velocity and accuracy are mean values ± standard deviations. Values for velocity categorization are absolute numbers. *F* Female; *M* Male

Irrespective of group, the correlation analyses yielded solely small associations between YBT-UQ performance and throwing velocity/accuracy (13-year-old females: − 0.01 ≤ *r* ≤ − 0.37 / 0.01 ≤ *r* ≤ 0.31; 14-year-old males: 0.10 ≤ *r* ≤ 0.45 / -0.01 ≤ r ≤ .-0.51; 15-year-old males: 0.06 ≤ *r* ≤ 0.34 / 0.01 ≤ *r* ≤ − 0.45).

## Discussion

The first purpose of this study was to assess whether there are side differences in UQ-YBT performance following the regular execution of a sport with a strong unilateral characteristic as given in Olympic handball. The second purpose was to determine whether a correlation exists between the UQ-YBT performance and the sport-specific measures (i.e. throwing accuracy and throwing velocity) of Olympic handball. The main results can be summarized as follows: (1) Overall, no consistent side differences could be detected for YBT-UQ performance of adolescent Olympic handball players in the present study. Side differences were only found in the 14-year-old male subjects; (2) Shoulder mobility/stability as assessed by the YBT-UQ did not predict shooting velocity and accuracy in adolescent Olympic handball players. The YBT-UQ was used due to its potential to expose asymmetries, which may lead to an increased risk of injury and functional decrements.

### Side differences in YBT-UQ performance

Due to the mainly unilateral characteristic of Olympic handball and the regular sport-specific Olympic handball training process, it was expected that side differences existed between the throwing and the non-throwing arm. However, these side differences could not be confirmed in the present study. This finding goes in line with previous studies [34–36] on athletes performing a mainly unilateral sport. However, these studies mainly focused on high school and college baseball players, and sports in which less throws are performed with a ball with substantially less weight than an Olympic handball.

The side differences that were found in the 14-year-old male subjects in SL reach direction are a rather surprising finding, given that no differences were found in either direction in the 13-year-old female and 15-year-old male subjects. However, Wilson et al. [37] also reported a significant reach difference between the throwing arm and the non-throwing arm in SL reach direction in water polo players. Possibly, the better results of the non-throwing arm as the stance arm could be the result of its stabilising function and adaptation following throws with the throwing arm [37]. Therefore, the throwing arm may profit from a higher mobility whereas the non-throwing arm may adapt with greater stabilizing abilities following repetitive throws and passes. Additionally, the SL reach direction is the one most closely resembling the typical throwing movement [37]. Thus, it could be concluded that a significant functional adaptation in terms of asymmetries in the remaining directions does not occur possibly due to the overall training load for the adolescent Olympic handball players not being sufficient to lead to these adaptations.

### Associations between YBT-UQ and throwing performance

Based on the present findings, shoulder mobility/stability as assessed by the YBT-UQ did not predict shooting velocity and accuracy in adolescent Olympic handball players. The finding that YBT-UQ and throwing performance solely showed small correlations goes in line with Štirn et al. [38] who reported a high number of factors contributing to the final ball velocity. Therefore, throwing was described as a highly multi-factorial skill, which cannot be predicted only based on the assessment of stability and mobility of the upper extremities [38]. Eriksrud [14] also reported no correlation between mobility and stability values as assessed through the hand reach star excursion balance test and throwing velocity in elite female Olympic handball players.

Olympic handball could be hypothesized as having an even stronger unilateral characteristic as other mainly unilaterally executed sports like basketball and soccer, due to the fact that passing and throwing are seldom executed with the non-throwing arm. Contrary to baseball or soccer, techniques in Olympic handball, which will also lead to functional adaptation [3] such as blocks, tackles in defence and any form of body contact are executed with both arms in equal distribution. This may be an explanation for the non-existing differences between the throwing and non-throwing arm. Olympic handball is therefore a sport with a mixture of closed and open chain actions. Probably, the rather low weight of the ball (ranging from 290 to 375 g for adolescents), albeit the high number of passes and throws, is not a sufficient stimulus to lead to significantly more pronounced strength and mobility adaptations of the throwing side.

Variations of the throwing technique with the elbow being shoulder height or higher may have led to different adaptations. Throwing velocity seems to be more important for backcourt players [39] who often have to throw from outside 9 m whereas accuracy seems to be more important for the wingers and pivots. A differentiation between playing positions could therefore be a valuable approach when assessing throwing velocity and accuracy. From a tactical point of view, it should also be kept in mind that fast executed throws in the game situation can be more important than high throwing velocities. The present study was executed from a central position, whereas several throws in the game are executed from different angles including the wings and only up to 10% of the throws in the game are standing throws with no run-up [15]. Therefore, future studies could also include jump throws, which are frequently displayed in training and games.

### Limitations

There are a few limitations of this study that need to be outlined. First, as long as the requirement of the elbow being at least shoulder height during the throwing, variations of the throwing technique were possible. With this regimen, we followed the procedure of the German Handball Federation [30] in order to achieve comparable results. Second, the throwing was executed without contact of an opponent or the cognitive component of decision making which is always present in games, especially when goalkeepers and defenders have to be surpassed. Third, the present results can only be applied to standing throws and therefore the results cannot be transferred to the other throwing techniques, e.g., with a run-up or jump throws.

## Conclusions

Olympic handball is a sport with a strong unilateral characteristic. The expected differences between the throwing and the non-throwing arm could not be confirmed in the present study. The lack of side differences leads to the possible consequence that the measurement of one side in all reach directions may be sufficient in determining stability and mobility of the upper extremities in adolescent Olympic handball players. Further and also against our assumption, YBT-UQ and throwing performance only showed a small predictive value. Therefore, alternative tests should be developed to predict throwing performances in adolescent Olympic handball players.

## Data Availability

The data generated and analyzed during the present study are not publicly available due to ethical restrictions but are available from the corresponding author upon reasonable request.

## Abbreviations

pts.: Points
YBT-UQ: Upper quarter Y balance test
SL: Superolateral
MED: Medial
IF: Inferolateral
M: Male
F: Female
te: Training experience
